# Photoinduced Biohydrogen Production from Biomass

**DOI:** 10.3390/ijms9071156

**Published:** 2008-07-08

**Authors:** Yutaka Amao

**Affiliations:** Department of Applied Chemistry, Oita University Dannoharu 700, Oita 870-1192, Japan E-Mail: amao@cc.oita-u.ac.jp; Tel. +81-97-554-7972

**Keywords:** biohydrogen, biomass, enzymatic hydrolysis, photoenergy, photosynthesis, saccharide

## Abstract

Photoinduced biohydrogen production systems, coupling saccharaides biomass such as sucrose, maltose, cellobiose, cellulose, or saccharides mixture hydrolysis by enzymes and glucose dehydrogenase (GDH), and hydrogen production with platinum colloid as a catalyst using the visible light-induced photosensitization of Mg chlorophyll-*a* (Mg Chl-*a*) from higher green plant or artificial chlorophyll analog, zinc porphyrin, are introduced.

## 1. Introduction

**Figure 1.**
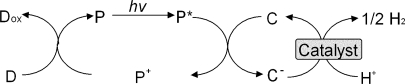
Photoinduced hydrogen production system consisting of an electron donor (D), a photosensitizer (P), an electron carrier (C) and a hydrogen producing catalyst.

Photoinduced hydrogen production systems have been studied extensively by means of converting solar energy to chemical energy [1–4]. Photoinduced hydrogen production systems consist of electron donor, photosensitizer, electron carrier and catalyst for hydrogen production as shown in Figure 1. For hydrogen evolved catalyst, platinum colloid and hydrogenase from *Desulfovibrio vulgaris* (Miyazaki) are widely used in hydrogen production systems [5–20]. Especially, platinum colloid is stable against long-term irradiation [9–11]. In photoinduced hydrogen production system with visible light, as water-soluble zinc porphyrins have absorption band in the visible light region (380–600nm), these porphyrins have been widely used as an effective photosensitizer. Especially, zinc tetraphenylporphyrin tetrasulfonate (ZnTPPS) is useful as a photosensitizer [17, 18]. However, themolar absorption coefficient of zinc porphyrins in the visible light region (500–600nm) is lower than that in the near ultra-visible light region (380–400nm).

In contrast, chlorophylls and bacteriochlorophylls act as the important roles of light-harvesting, photoinduced energy transfer and photoinduced charge separation functions in photosynthesis reactions [21]. There photosynthesis dyes have the absorption maxima at 670–800nm. Moreover, themolar absorption coefficient of these dyes is larger than that of zinc porphyrin, the modelmolecules for chlorophyll. The photoinduced hydrogen production system using the photosensitization of chlorophyllmolecule also developed [22–25]. As mentioned above, the photoinduced hydrogen production with the system consisting of electron donor, photosensitizer, electron carrier and catalyst are widely studied. In this system, however, electron donor is a sacrificial reagent and the oxidized electron donor is consumed in the reaction system and the hydrogen production is stopped with consumption of electron donor. Moreover, NADH or NADPH, which is an effective electron donor in photoinduced hydrogen production system, is an expensive reagent and the system with NADH or NADPHas a sacrificial reagent is not desirable. However, NADH or NADPHcan be regenerated, the photoinduced hydrogen production system would be accomplished without NAD^+^ or NADP^+^ consumption. Since glucose dehydrogenase (GDH) uses NAD^+^ as a cofactor, a photoinduced hydrogen production can be developed using the combination of two reactions. One is the NADH regeneration with GDH and the other one is hydrogen production system with photosensitizer, electron relay reagent, and catalyst. Some renewable biomass resources are starch, cellulose, sucrose, lactose, and so on. These oligosaccharides and polysaccharides are hydrolyzed to form monosaccharides such as glucose. The conversion of glucose to hydrogen will be a useful new enzymatic pathway. Some studies on the hydrogen production from glucose using the enzymatic and biological pathway have been reported [26–33]. Moreover, the development of hydrogen production system using solar energy and the saccharide biomass is innovative.

In this review, the visible-light induced biohydrogen production systems, coupling saccharides biomass such as sucrose, maltose, cellobiose, cellulose, or saccharides mixture hydrolysis by enzymes and glucose dehydrogenase (GDH), and hydrogen production with platinum colloid as a catalyst using the visible light-induced photosensitization of Mg chlorophyll-*a* (Mg Chl-*a*) or artificial chlorophyll analog, water-soluble zinc porphyrin, are introduced.

## 2. Principle of photoinduced biohydrogen production from saccharide biomass

**Figure 2.**
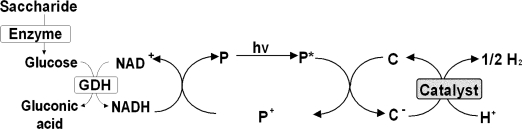
Photoinduced biohydrogen production system with the combination of NADH production with saccharide hydrolysis by enzyme and GDH and hydrogen production consisting of NADH, a photosensitizer (P), an electron carrier (C) and a hydrogen producing catalyst.

The photoinduced biohydrogen production from saccharide biomass is shown in Figure 2. This system consists of two reaction systems. One is the saccharide hydrolysis with hydrolysis functional enzyme and NADH production with GDH. The other is the photoinduced hydrogen production with the system containing a photosensitizer (P) such as Mg Chl-*a* or water-soluble zinc porphyrin, an electron carrier (C) such as methylviologen (MV^2+^), a hydrogen producing catalyst such as platinum colloid and an electron donor NADH. The two reactions are connected via the NAD^+^/NADH redox system.

## 3. Photoinduced biohydrogen production from glucose with GDH, NAD+, zinc porphyrin, methylviologen and hydrogenase system

Although in theory the amount of hydrogen that could be generated from renewable sources of energy such as cellulose is vast, only 16–24% of the maximum stoichiometric yield of hydrogen from glucose (about 12mol hydrogen permol glucose) is typically achieved by biological methods. The enzymes of the oxidative pentose phosphate cycle can be coupled to hydrogenase purified from the bacterium *Pyrococcus furiosus*, one of only a few hydrogenases that use NADP^+^ as the electron carrier, to generate 11.6mol hydrogen permol glucose-6-phosphate. Hydrogen produced by this pathway is the major product, unlike that produced by intermediate metabolic pathways of bacterial fermentation, and therefore has important practical implications for biohydrogen production [34].

**Figure 3.**
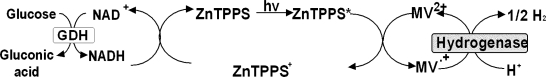
Hydrogen production system consisting of glucose, GDH, NAD^+^ and hydrogenase.

Another hydrogen production system from glucose consisting of GDH and hydrogenase from *Alcaligenes eutrophus* H16, mesophilic hydrogen oxidizing bacterium, is introduced [35]. The reaction system is shown in Figure 3. The production of hydrogen from glucose is carried out as follows. The reaction mixture (4.1ml) containing glucose (2.1mmol), GDH (10units), and hydrogenase (4.5units) in 5ml test tube is sealed and is deaerated by freeze pump thaw cycle for 3 times, and by flush with N_2_. Oneunit of GDH activity is defined as the amount of enzyme that reduced 1.0μmol NAD^+^ to NADH by glucose permin. Oneunit of hydrogenase activity is defined as release of 1.0μmol of hydrogen permin.. The reaction is started by adding N_2_ saturated 4.1μmol NAD^+^ solution to the reaction mixture. When the solution containing hydrogenase (2.2units) and NADH (1.2mmol) is incubated at 30 °C under N_2_ at pH7.0, 70μmol hydrogen is evolved after 40min incubation confirming the hydrogen production from NADH with the hydrogenase from *Alcaligenes eutrophus* H16. The hydrogenase finds to catalyze the hydrogen evolution from NADH stoichiometrically, indicating that no side reaction of NADH oxidation occurs. When the solution containing glucose (2.1mmol), GDH (10units), NAD^+^ (4.1μmol), and hydrogenase (4.5units) is incubated at 30°C (pH7.0), about 3.0μmol of hydrogen is evolved after 1h incubation. This demonstrates the hydrogen production system from glucose consisting of GDH, NAD^+^, and hydrogenase as shown in Figure 3.

**Figure 4.**
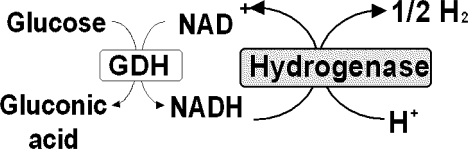
Photoinduced biohydrogen production from glucose with artificial chlorophyll analogmolecule ZnTPPS-hydrogenase system.

Next, photoinduced biohydrogen production from glucose with GDH, NAD^+^, water-soluble zinc porphyrin, methylviologen (MV^2+^) and hydrogenase system is introduced [36]. The reaction system is shown in Figure 4. Photoreduction of MV^2+^ is carried out as follows. The reaction mixture (3ml, pH8.0) containing tris(hydroxymethyl)-aminomethane (Tris, 150μmol), glucose (990μmol). GDH (39units), NAD^+^ (3μmol). ZnTPPS (2.2nmol) and MV^2+^ (900nmol) is deaerated, by freeze pump thaw cycles and by flush with Ar in a glass vessel. After deaeration, the reaction mixture is irradiated using 200W tungsten lamp through 390nm cut off filter. The reduction of MV^2+^ is measured by UV spectrometer at 600nm. When the solution containing glucose, GDH, NAD^+^, ZnTPPS, and MV^2+^ is irradiated under anaerobic conditions, the absorbance at 600nm typical absorption band of reduced form of MV^2+^, increases. After 40min irradiation, 0.06mmol dm^−3^ MV^2+^ is photoreduced.

The production of hydrogen from glucose is carried out as follows. The reaction mixture (3.0ml, pH8.0) containing Tris (150μmol), glucose (990μmol), GDH (39units), NAD^+^ (3.0μmol), ZnTPPS (2.2nmol), MV^2+^ (900nmol) and hydrogenase (0.6units) is deaerated by freeze pump thaw cycle for 3 times, and by flush with N_2_. The reaction is started by irradiation as mentioned above. Produced hydrogen is measured by gas chromatograpHwith N_2_ carrier. By irradiation, hydrogen evolves continuously more than 9h. After 8hirradiation, 2.5μmol hydrogen evolves. Thus the photoinduced hydrogen evolution system, which contains glucose as the electron donor as shown in Figure 4, is established.

## 4. Photoinduced biohydrogen production with sucrose hydrolysis and chlorophyll-platinum colloid system

**Figure 5.**
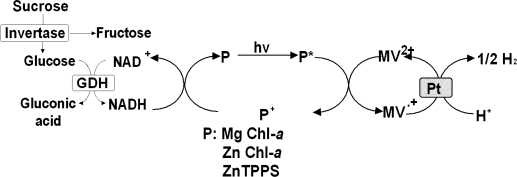
Photoinduced biohydrogen production with sucrose hydrolysis and chlorophyll or artificial chlorophyll analogmolecule-platinum colloid system.

Sucrose is disaccharide and consists of glucose and fructose. Sucrose is hydrolyzed into glucose and fructose with invertase as shown in Figure 5. In this section, the photoinduced biohydrogen production with the system consisting of sucrose hydrolysis with invertase and hydrogen production with chlorophyll-platinum colloid is introduced [37–39]. At first, NADH formation with the system consisting of sucrose, invertase, GDH, NAD^+^ is indicated. The conversion yield of NAD^+^ to NADH is *c.a* 100 % with 80min incubation at 30°C under the condition of NAD^+^ (0.80μmol), sucrose (16μmol), invertase (4.0units) and GDH (5.0units) in 3.0ml of 10mmol dm^−3^ potassium phosphate buffer (pH7.0). In contrast, no formation of NADH is observed in a solution containing sucrose, GDH and NAD^+^ without invertase. Thus, NADH is formed via sucrose hydrolysis with invertase.

Next, the photoreduction of MV^2+^ with the system containing NAD^+^, sucrose, Mg Chl-*a*, MV^2+^, invertase and GDH with visible light irradiation is introduced. The absorbance at 605nm, absorption band of MV^+^, increases with irradiation time. After 80min irradiation, 0.2μmol reduced MV^2+^ is produced and the yield of MV^2+^ to MV^.+^ is estimated to be *c.a.* 33% under the condition of NAD^+^ (15μmol), sucrose (0.30mmol), Mg Chl-*a* (3.0nmol), MV^2+^ (1.2μmol), invertase (4.0units) and GDH (5.0units) in 3.0ml of 10mmol dm^−3^ potassium phosphate buffer (pH7.0). Oneunit of invertase activity is defined as the amount of enzyme that produced 1.0μmol glucose by sucrose permin. The sample solution is deaerated by repeated freeze-pump-thaw cycles and irradiated with a 200W tungsten lamp at a distance of 3.0cm, with a light intensity of 200J m^−2^ s^−1^, at 30°C. The light of the wavelength less than 390nm is removed by Toshiba L-39 cut-off filter. The photoreduction rate is independent of the concentrations of NAD^+^, sucrose, invertase and GDH. On the other hand, the reduction rate depends on the concentrations of Mg Chl-*a* and MV^2+^. Thus, the rate-limiting step in the MV^2+^ reduction is the photoinduced electron transfer process from the photoexcited Mg Chl-*a* (*Mg Chl-*a*) to MV^2+^. On the other hand, MV^2+^ is not reduced without NAD^+^ in the above system. There is no direct electron transfer between sucrose or glucose formed with invertase and MV^2+^, and between Mg Chl-*a* and MV^2+^. Thus, the visible light-induced MV^2+^ reduction proceeds by coupling the sucrose hydrolysis with invertase and GDH and MV^2+^ reduction using the photosensitization of Mg Chl-*a*.

As the MV^2+^ photoreduction system containing NAD^+^, sucrose, Mg Chl-*a*, MV^2+^, invertase and GDH is achieved, the photoinduced hydrogen production system is introduced. The photoinduced hydrogen production from sucrose is carried out as follows. The sample solution containing NAD^+^ (15μmol), sucrose (0.30mmol), Mg Chl-*a* (3.0nmol), MV^2+^ (1.2μmol), platinum colloid (0.5unit), invertase (4.0units) and GDH (5.0units) in 3.0mol of 10mmol dm^−3^ potassium phosphate buffer (pH7.0) is deaerated by freeze pump thaw cycle for 6 times, and is substituted by argon gas. One unit of platinum colloid activity is defined as release of 1.0μmol of hydrogen per min. The amount of hydrogen evolved is measured by gas chromatography (detector: TCD, column temperature: 4°C, column: active charcoal with the particle size 60~80 mesh, carrier gas: nitrogen gas, carrier gas flow rate: 24ml min^−1^). By irradiation, hydrogen evolves continuously more than 420min. The amount of hydrogen production is estimated to be 4.3 μmol after 420min irradiation. It indicates that 8.6 μmol of proton, that is 2867 times the amount of Mg Chl-*a* (3.0nmol) in the sample solution, is reduced to hydrogenmolecules. Therefore, the Mg Chl-*a* appears to serve as the system for transferring electrons from NADH, which is formed from sucrose, to a more reductive hydrogenmolecule. On the other hand, the hydrogen also is not evolved in the absence of NAD^+^ in the above system. There is no direct electron transfer between sucrose or glucose formed with invertase and platinum colloid, and among Mg Chl-*a*, MV^2+^ and platinum colloid. However, the amount of hydrogen gas evolved is less than the initial amount of NAD^+^ added within 420min irradiation. Thus, the hydrogen production is attempted with long-term irradiation. After 48hcontinuously irradiation, the amount of hydrogen production is estimated to be 23 μmol. As the amount of hydrogen gas evolved is more than the initial amount of NAD^+^ added with long term irradiation, therefore, NADH is recycle to NAD^+^. These results suggest that the visible light-induced hydrogen production proceeded by coupling the sucrose hydrolysis with invertase and GDH and the hydrogen production with platinum colloid using the photosensitization of Mg Chl-*a*.

In general, Mg Chl-*a* purified is unstable against the irradiation. Recently, zinc bacteriochlorophyll-*a* is founded in an aerobic bacterium *Acidiphilium rubrum*[40–42]. As the zinc porphyrins are stable against the irradiation and effective photosensitizers, zinc chlorophylls also are attractive compound as a stable visible photosensitizer. Thus, the enzymatic photoinduced hydrogen productions from sucrose using the photosensitization of Zn chlorophyll-*a* (Zn Chl-*a*) [43–45] and water-soluble zinc porphyrin ZnTPPS [46, 47] is introduced. Photoreduction of MV^2+^ is tested in the reaction mixture containing NAD^+^, sucrose, Zn Chl-*a*, MV^2+^, invertase and GDH. The reaction system consists of NAD^+^ (15μmol), sucrose (0.30mmol), Zn Chl-*a* (4.5nmol), MV^2+^ (1.2μmol), invertase (4.0units) and GDH (5.0units) in 3.0ml of 10mmol dm^−3^ potassium phosphate buffer (pH7.0). The absorbance at 605nm, absorption band of MV^.+^, increases with irradiation time. After 80min irradiation, 0.69μmol reduced MV^2+^ is produced and the yield of MV^2+^ to MV^.+^ is estimated to be *c.a.* 58%. For the system using Mg Chl-*a*, 0.21μmol reduced MV^2+^ is produced and the yield of MV^2+^ to MV^.+^ is estimated to be *c.a.* 33% after 80min irradiation. Thus, the photosensitization activity of Zn Chl-*a* is superior to that of Mg Chl-*a*.

The photoinduced hydrogen production from sucrose is carried out under condition with the sample solution containing NAD^+^ (15μmol), sucrose (0.30mmol), Zn Chl-*a* (4.5nmol), MV^2+^ (1.2μmol), platinum colloid (0.5unit), invertase (4.0units) and GDH (5.0units) in 3.0ml of 10mmol dm^−3^ potassium phosphate buffer (pH7.0). By irradiation, hydrogen evolves continuously more than 4h. The amount of hydrogen production is estimated to be 7.0 μmol after 4hirradiation. It indicates that 14 μmol of proton, that is 3111 times the amount of Zn Chl-*a* (4.5nmol) in the sample solution, is reduced to hydrogenmolecules. Therefore, the Zn Chl-*a* appears to serve as the system for transferring electrons from NADH, which is formed from sucrose, to a more reductive hydrogenmolecule. For the system using Mg Chl-*a*, the amount of hydrogen production is estimated to be 3.0μmol after 4hirradiation. It indicated that 6.0μmol of proton, that is 2000 times the amount of Mg Chl-*a* (3.0nmol) in the sample solution, is reduced to hydrogenmolecules. Moreover, the amount of hydrogen production is estimated to be 68μmol after 48h continuously irradiation. In this system, the amount of hydrogen gas evolved also is more than the initial amount of NAD^+^ added with long term irradiation. Thus, the Zn Chl-*a* has highly activity of photosensitization compared with that of Mg Chl-*a* in the photoinduced hydrogen production system.

Next, the photoinduced hydrogen production with the combination of sucrose hydrolysis and the system with ZnTPPS-platinum colloid is introduced. When the deaerated sample solution containing NAD^+^ (15μmol), sucrose (0.30mmol), ZnTPPS (3.0nmol), MV^2+^ (1.2μmol), invertase (4.0units) and GDH (5.0units) in 3.0ml of 10mmol dm^−3^ phosphate buffer (pH=7.0) is irradiated, the absorbance at 605nm, absorption band of reduced MV^2+^, increases with irradiation time. After 180min irradiation, 1.1μmol reduced MV^2+^ is produced. Thus, the conversion of MV^2+^ to the reduced MV^2+^ is *c.a.* 100% after 180min irradiation.

As the MV^2+^ photoreduction system containing sucrose as an electron-donating reagent is achieved, the development of photoinduced hydrogen production system is introduced. The photoinduced hydrogen production from sucrose is carried out as follows. The sample solution containing sucrose (0.30mmol), ZnTPPS (3.0nmol), MV^2+^ (1.2μmol), platinum colloid (0.12μmol), invertase (4.0units) and GDH (5.0units) in 10mmol dm^−3^ phosphate buffer (pH=7.0) is deaerated by freeze pump thaw cycle for 6 times, and substituted by argon gas. The NAD^+^ (15μmol) solution, which is deaerated and substituted by argon gas, is added to the above solution and then the reaction is started by the irradiation. By irradiation, hydrogen evolves continuously more than 7h. The amount of hydrogen production is estimated to be *c.a.* 18μmol after 7hirradiation. In this system, the amount of hydrogen gas evolved also is more than the initial amount of NAD^+^ added with 7hirradiation. These results are summarized in Tables 1 and 2.

## 5. Photoinduced biohydrogen production with D-maltose hydrolysis and chlorophyll-platinum colloid system

**Figure 6.**
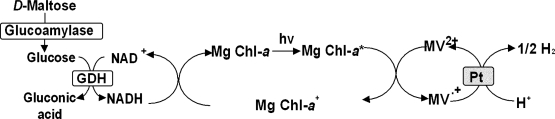
Photoinduced biohydrogen production with *D*-maltose hydrolysis and chlorophyll-platinum colloid system.

*D*-Maltose is a disaccharide formed from twounits of glucose joined with an α (1→4) linkage. Maltose is broken down into two glucosemolecules by hydrolysis. In living organisms, the enzyme maltase or glucoamylase achieve hydrolysis very rapidly as shown in Figure 6. In this section, the photoinduced biohydrogen production with the system consisting of *D*-maltose hydrolysis with glucoamylase and hydrogen production with chlorophyll-platinum colloid is introduced [48, 49]. NADH formation with the system consisting of *D*-maltose, glucoamylase, GDH, NAD^+^ is indicated. The reaction condition is NAD^+^ (6.0μmol), *D*-maltose (11μmol), glucoamylase and GDH (5.0units) in 3.0ml of 10mmol dm^−3^ potassium phosphate buffer (pH7.0). Oneunit of glucoamylase activity is defined as the amount of enzyme that produced 1.0μmol glucose by maltose permin. The initial rate of NADH formation is determined by the amount of NADH after incubation for 10min. The rate of formation increases with the concentration of glucoamylase up to 8.0units and then became constant value. In contrast, no formation of NADH is observed in a solution containing *D*-maltose, GDH and NAD^+^. Thus, NADH is formed via *D*-maltose hydrolysis with glucoamylase. Eightunits of glucoamylase, formed 0.6μmol NADH after 120min under above condition. The yield of conversion of NAD^+^ to NADH in this system is almost 100%.

Next, the photoreduction of MV^2+^ with the system containing NAD^+^, *D*-maltose, Mg Chl-*a*, MV^2+^, glucoamylase and GDH with visible light irradiation is introduced. The reaction condition is NAD^+^, *D*-maltose (0.30mmol), Mg Chl-*a* (3.0nmol), MV^2+^ (1.2μmol), glucoamylase (8.0units) and GDH (5.0units) in 3.0ml of 10mmol dm^−3^ potassium phosphate buffer (pH7.0). The absorbance at 605nm attributed to the absorption band of MV^.+^ also increases with irradiation time. The rate of formation increases with the concentration of NAD^+^ up to 15μmol and then the rate decreases. After 40min irradiation, 0.18μmol □of reduced MV^2+^ is produced and the yield of MV^2+^ to MV^.+^ is estimated to be about 15 %. The photoreduction rate is independent of the concentrations of *D*-maltose, glucoamylase and GDH. In contrast, MV^2+^ is not reduced without NAD^+^ in the system. There is no direct electron transfer between *D*-maltose or glucose formed with glucoamylase and MV^2+^, and between Mg Chl-*a* and MV^2+^. Thus, the visible light-induced MV^2+^ reduction proceeds by the coupling of *D*-maltose hydrolysis with glucoamylase and GDH and MV^2+^ reduction using the photosensitization of Mg Chl-*a*.

Photoinduced hydrogen production with the system containing NAD^+^, *D*-maltose, Mg Chl-*a*, MV^2+^, glucoamylase, GDH and platinum colloid is introduced. The reaction condition is NAD^+^ (15μmol), *D*-maltose (0.30mmol), Mg Chl-*a* (3.0nmol), MV^2+^ (1.2μmol), platinum colloid (0.5unit), glucoamylase (8.0units) and GDH (5.0units) in 3.0ml of 10mmol dm^−3^ potassium phosphate buffer (pH7.0). Hydrogen is produced continuously for more than 4hunder visible light irradiation. The amount of hydrogen production is estimated to be 5.0μmol after 4h. The conversion of *D*-maltose to hydrogen gas is about 1.8%. On the other hand, hydrogen is not produced in the absence of NAD^+^ in the above system. There is no direct electron transfer between *D*-maltose or glucose formed with glucoamylase and Pt colloid, and Mg Chl-*a*, MV^2+^ and platinum colloid. Moreover, the amount of hydrogen production is estimated to be 50μmol after 48h continuously irradiation. In this system, the amount of hydrogen gas evolved also is more than the initial amount of NAD^+^ added with long term irradiation. These results suggest that the visible light-induced hydrogen production proceeded by coupling *D*-maltose hydrolysis with glucoamylase and GDH and hydrogen production with platinum colloid using photosensitization of Mg Chl-*a*.

## 6. Photoinduced biohydrogen production with cellulose derivative hydrolysis and chlorophyll-platinum colloid system

**Figure 7.**
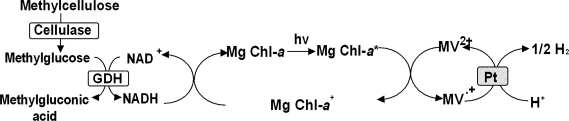
Photoinduced biohydrogen production with cellulose derivative methylcellulose hydrolysis and chlorophyll-platinum colloid system.

Exploitation of energy of cellulose is desired, because cellulose is the main ingredients, such as wood waste. Cellulose is a polysaccharide consisting of a linear chain of several hundred to over ten thousand β (1→4) linked *D*-glucoseunits. Cellulose is the structural component of the primary cell wall of green plants. Cellulose is broken down into two glucosemolecules by hydrolysis with cellulase as shown in Figure 7. To attain the photoinduced biohydrogen production from cellulose, it is necessary to develop the solubilization of cellulose in water media. In this section, the photoinduced hydrogen production system by coupling the water-soluble cellulose derivative, methylcellulose, hydrolysis with cellulase and GDH, and hydrogen production with platinum colloid via the photoreduction of MV^2+^ using the sensitization of Mg Chl-*a* is introduced[50,51]. NADH formation with the system consisting of methylcellulose, cellulase, GDH, NAD^+^ is indicated. The reaction condition is NAD^+^ (0.52μmol) to a solution containing methylcellulose (1.1μmol), cellulase and GDH (5.0units) in 3.0ml of 10mmol dm^−3^ potassium phosphate buffer (pH7.0). Oneunit of cellulase activity is defined as the amount of enzyme that produced 1.0μmol glucose by methylcellulose permin. The initial rate of NADH formation is determined by the amount of NADH after incubation for 10min. The rate of formation increases with the concentration of cellulase up to 4.0units and then becomes constant value. In contrast, no formation of NADH is observed in a solution containing methylcellulose, GDH and NAD^+^. Thus, NADH is formed via methylcellulose hydrolysis with cellulase. Fourunits of cellulase, formed 0.52μmol NADH after 40min. The yield of conversion of NAD^+^ to NADH in this system is almost 100%.

Next, the photoreduction of MV^2+^ with the system containing NAD^+^, methylcellulose, Mg Chl-*a*, MV^2+^, cellulase and GDH with visible light irradiation is introduced. The absorbance at 605nm, absorption band of MV^+^, increases with irradiation time. After 50min irradiation, 0.3 μmol reduced MV^2+^ is produced and the yield of MV^2+^ to MV^.+^ is estimated to be *c.a.* 25% under the condition of NAD^+^ (15 μmol), methylcellulose (30μmol monomerunit), cellulase (4.0units) and GDH (5.0units), Mg Chl-*a* (3.0nmol), and MV^2+^ (1.2μmol) in 3.0ml of 10mmol dm^−3^ potassium phosphate buffer (pH7.0). The photoreduction rate is independent of the concentrations of NAD^+^, methylcellulose, cellulase and GDH. In contrast, the reduction rate depends on the concentrations of Mg Chl-*a* and MV^2+^. Thus, the rate-limiting step in the MV^2+^ reduction is the electron transfer process from the photoexcited Mg Chl-*a* (*Mg Chl-*a*) to MV^2+^. On the other hand, MV^2+^ is not reduced without NAD^+^ in the above system. Thus, the MV^2+^ photoreduction proceeds by coupling the methylcellulose hydrolysis with cellulase and GDH and MV^2+^ reduction using the photosensitization of Mg Chl-*a*.

The photoinduced hydrogen production in the system containing methylcellulose, cellulase, GDH, NAD^+^, Mg Chl-*a*, MV^2+^, and platinum colloid by the visible light is introduced. The reaction condition is NAD^+^ (15μmol), methylcellulose (30μmol monomerunit), cellulase (4.0units), GDH (5.0units), Mg Chl-*a* (3.0nmol), MV^2+^ (1.2μmol) and platinum colloid (0.5unit) in 3.0ml of 10mmol dm^−3^ potassium phosphate buffer (pH7.0). From the result of gases analysis in gaseous phase using chromatography, hydrogen and argon gases are detected and the other gases is not detected. Carbon dioxide gas also is not detected. The by-product formation in reaction mixture is analyzed using HPLC with an electrical conductivity detector (column temperature: 40 °C, column: polystyrene sulfonate column, elutant: *p*-toluene sulfonic acid and flow rate: 0.8mlmin^−1^). 6-Methylgluconic acid is produced as by-product. 6-Methylgluconic acid is formed by oxidation of 6-methylglucose (formed by methylcellulose hydrolysis with cellulase) with GDH. Thus, the visible light-induced hydrogen production proceeds by coupling the methylcellulose hydrolysis with cellulase and GDH and the hydrogen production with platinum colloid using the photosensitization of Mg Chl-*a*. By irradiation, hydrogen produces continuously more than 4h. The amount of hydrogen production is estimated to be 12 μmol after 4hirradiation. Moreover, the amount of hydrogen production is estimated to be 30 μmol after 10hcontinuously irradiation. In this system, the amount of hydrogen gas evolved also is more than the initial amount of NAD^+^ added with 10hirradiation. It indicates that 24μmol of proton, that is 8000 times the amount of Mg Chl-*a* (3.0nmol) in the sample solution, is reduced to hydrogenmolecules. Therefore, the Mg Chl-*a* appears to serve as the system for transferring electrons from NADH, which is formed from methylcellulose, to a more reductive hydrogenmolecule. On the other hand, the hydrogen also is not evolved in the absence of NAD^+^ in the above system. These results strongly suggest that the visible light-induced hydrogen production proceeded by coupling the methylcellulose hydrolysis with cellulase and GDH and the hydrogen production with platinum colloid using the photosensitization of Mg Chl-*a*.

Next let us focus on the relation among the degree of polymerization, molecular weight of methylcellulose and the amount of hydrogen production [52,53]. Methylcellulose (MC*_n_*) with averagemolecular weights of 15,000, 21,000, 26,000, 41,000, 63,000 and 86,000, are defined as the MC_1_, MC_2_, MC_3_, MC_4_, MC_5_ and MC_6_. When the sample solution containing MC*_n_* and cellulase is incubated for 10min, methylglucose (2-, 3- or 6-methylglucose) or glucose is formed. For all systems using MC*_n_*, 2.4 μmol methylglucose is produced. This result shows that the rate of MC*_n_* hydrolysis to methylglucose or glucose with cellulase is very rapid and the yield is *ca.* 100% after 10min incubation. When the sample solution containing MC*_n_*, cellulase, NAD^+^ and GDH is incubated, 0.6μmol NADH is formed and the yield of conversion of NAD^+^ to NADH by the MC*_n_* hydrolysis with cellulase and GDH is 100% after 40min incubation in all cases using MC*_n_*. Little change in NADH formation rate is observed for any MC*_n_*, and thus, MC*_n_* hydrolysis rate with cellulase is independent of the polymerization of MC*_n_*.

In all cases of MC*_n_*, 0.3 μmol reduced MV^2+^ is produced and the yield of MV^2+^ to MV^.+^ is estimated to be *c.a.* 25% under the condition of NAD^+^ (15 μmol), MC*_n_* (30 μmol monomerunit), cellulase (4.0units) and GDH (5.0units), Mg Chl-*a* (3.0nmol), and MV^2+^ (1.2μmol) in 3.0ml of 10mmol dm^−3^ potassium phosphate buffer (pH7.0). Little change in MV^2+^ photoreduction rate is observed for any MC*_n_*, and thus, MV^2+^ photoreduction rate is independent of the polymerization of MC*_n_*.

When the sample solution containing MC*_n_*, cellulase, GDH, NAD^+^, Mg Chl-*a*, MV^2+^, and platinum colloid is irradiated, the hydrogen production is observed in all systems using MC*_n_*. For the systems using MC_1_ and MC_4_, the amount of hydrogen production is 12μmol after 4hirradiation. In contrast, the amount of hydrogen production is 10μmol after 4hirradiation in the system using MC*_2_*. Moreover, the amount of hydrogen production is more than 25μmol after 10h continuously irradiation in all cases of MC*_n_*. Thus, the amount of hydrogen gas evolved also is more than the initial amount of NAD^+^ added with 10h irradiation. These results show that the hydrogen production rate also is independent of polymerization degree of MC*_n_*. These results are summarized in Tables 3 and 4.

## 7. Photoinduced biohydrogen production with saccharide mixture hydrolysis and chlorophyll-platinum colloid system

To apply this system for practical photoinduced biohydrogen production, it is necessary to accomplish the hydrogen production from various saccharides mixture biomass. In this section, the photoinduced biohydrogen production system by coupling three difference saccharaides mixture (sucrose, *D*-maltose and cellobiose) hydrolysis by three enzymes (invertase, glucoamylase and cellulase) and glucose dehydrogenase (GDH), and hydrogen production with platinum colloid as a catalyst using the visible light-induced photosensitization of Mg Chl-*a* is introduced[54]. When the sample solution containing NAD^+^ (0.33μmol), sucrose (6.0μmol), *D*-maltose (6.0μmol), cellobiose (6.0μmol), invertase (4.0units), glucoamylase (4.0units), cellulase (4.0units) and GDH (5.0units) in phosphate buffer (pH7.0) is incubated, 0.30μmol NADH is produced after 20min incubation. The reduction ratio of NAD^+^ to NADH is about 91%, showing that glucose formation from saccharides (sucrose, *D*-maltose and cellobiose) with the system of invertase, glucoamylase and cellulase proceeds rapidly.

When the sample solution containing NAD^+^ (15μmol), sucrose (12μmol), *D*-maltose (12μmol), cellobiose (9μmol), invertase (4.0units), glucoamylase (4.0units), cellulase (4.0units), GDH (5.0units), Mg Chl-*a* (13.5nmol), and MV^2+^(1.2μmol) in phosphate buffer (pH7.0) is irradiated, 0.24μmol MV^.+^ is produced after 60min irradiation,. Thus, the conversion of MV^2+^ to the reduced MV^+^ is about 20% after 60min irradiation. These results show that the MV^2+^ photoreduction proceeds by coupling the NADH formation due to the saccharides hydrolysis with the system of invertase, glucoamylase, cellulase, NAD^+^ and GDH and MV^2+^ reduction using the photosensitization of Mg Chl-*a*. As the MV^2+^ photoreduction system containing three different saccharides as an electron-donating reagent is achieved, the development of photoinduced hydrogen production system is attempted. By irradiation to the sample solution containing NAD^+^ (15μmol), sucrose (12μmol), *D*-maltose (12μmol ^3^), cellobiose (9μmol), invertase (4.0units), glucoamylase (4.0units), cellulase (4.0units), GDH (5.0units), Mg Chl-*a* (13.5nmol), MV^2+^ (1.2μmol) and platinum colloid (0.5unit) in phosphate buffer (pH7.0), hydrogen evolves continuously more than 2h. The amount of hydrogen production is estimated to be about 2.9μmol after 2h irradiation. As the amount of initial saccharides is 33μmol, the yield of saccharides to hydrogen gas is estimated to be 8.8% after 2h irradiation. The by-product formation in reaction mixture is analyzed using HPLC. Gluconic acid is produced as by-product and the amount of gluconic acid is 5.7μmol after 2h irradiation. Gluconic acid is formed by oxidation of glucose (formed by cellobiose, sucrose and maltose hydrolysis) with GDH. On the other hand, hydrogen also is not evolved in the absence of NAD^+^ or without irradiation condition. There is no direct electron transfer between three saccharides or glucose formed with three enzymes and platinum colloid, and among Mg Chl-*a*, MV^2+^ and platinum colloid. Moreover, the amount of hydrogen production is estimated to be 57μmol after 48h continuously irradiation. In this system, the amount of hydrogen gas evolved also is more than the initial amount of NAD^+^ added with long term irradiation. These results suggest that the visible light-induced hydrogen production proceeded by coupling the three different saccharides hydrolysis with three enzymes and GDH and the hydrogen production with platinum colloid using the photosensitization of Mg Chl-*a*. The results of MV^2+^ photoreduction and hydrogen production from various saccharides are summarized in Tables 5 and 6.

## 8. Conclusion

In this review, green process for hydrogen production system coupling saccharide biomass hydrolysis with hydrolysis functional enzyme and GDH and hydrogen production with platinum colloid via MV^2+^ photoreduction using light-harvesting function of Mg Chl-*a* or its analogs is developed and the continuous hydrogen gas is achieved. Gluconic acid from saccharide biomass only is produced as a by-product and no carbon dioxide gas is evolved in present reaction system. Thus, renewable bio-resources have been effectively used to produce, hydrogen gas through on environmentally clean process.

## Figures and Tables

**Figure 8.**
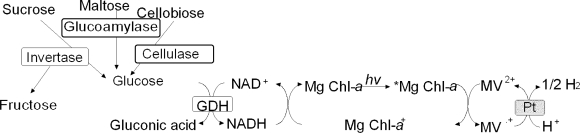
Photoinduced biohydrogen production with saccharide mixture consisting of *D*-maltose, sucrose and cellobiose hydrolysis and chlorophyll-platinum colloid system.

**Table 1.** Reduced MV^2+^ (MV^.+^) production rate with the system of sucrose, invertase, GDH, NAD^+^, photosensitizer and MV^2+^.

Photosensitizer	Rate of MV^.+^ production /μmolmin^−1^	Reference
Mg Chl-*a*	0.0025	[37–39]
Zn Chl-*a*	0.0086	[43–45]
ZnTPPS	0.014	[46, 47]

**Table 2.** Hydrogen production rate with the system of sucrose, invertase, GDH, NAD^+^, photosensitizer, MV^2+^ and platinum colloid.

Photosensitizer	Rate of hydrogen production/μmol h^−1^	Reference
Mg Chl-*a*	0.61	[37–39]
Zn Chl-*a*	1.75	[43–45]
ZnTPPS	2.57	[46, 47]

**Table 3.** Reduced MV^2+^ (MV^.+^) production rate with the system of methylcellulose, cellulase, GDH, NAD^+^, Mg Chl-*a* and MV^2+^.

Molecular weight of methylcellulose	Rate of MV^.+^ production/μmolmin^−1^
15,000 (MC_1_)	0.0060
21,000 (MC_2_)	0.0061
26,000 (MC_3_)	0.0058
41,000 (MC_4_)	0.0060
63,000 (MC_5_)	0.0060
86,000 (MC_6_)	0.0059

**Table 4.** Hydrogen production rate with the system of methylcellulose, cellulase, GDH, NAD^+^, Mg Chl-*a*, MV^2+^ and platinum colloid.

Molecular weight of methylcellulose	Rate of hydrogen production/μmol h^−1^
15,000 (MC_1_)	2.9
21,000 (MC_2_)	3.0
26,000 (MC_3_)	2.3
41,000 (MC_4_)	3.0
63,000 (MC_5_)	2.9
86,000 (MC_6_)	2.8

**Table 5.** Reduced MV^2+^ (MV^.+^) production rate with the system of saccharide, hydrolysis enzyme, GDH, NAD^+^, Mg Chl-*a* and MV^2+^.

Saccharide	Rate of MV^.+^ production/μmolmin^−1^	Reference
*D-*Maltose	0.0045	[48,49]
Methylcellulose	0.0060	[50,51]
Mixture of sucrose, *D*-maltose and cellobiose	0.0040	[54]

**Table 6.** Hydrogen production rate with the system of saccharide, hydrolysis enzyme, GDH, NAD^+^, Mg Chl-*a*, MV^2+^ and platinum colloid.

Saccharide	Rate of hydrogen production/μmol h^−1^	Reference
*D*-Maltose	1.25	[48,49]
Methylcellulose	3.0	[50,51]
Mixture of sucrose, *D*-maltose and cellobiose	1.45	[54]
